# Class inequalities in physical functioning trajectories before and after retirement

**DOI:** 10.1093/eurpub/ckac131.195

**Published:** 2022-10-25

**Authors:** J Lahti, O Pietiläinen, O Rahkonen, T Lallukka

**Affiliations:** Department of Public Health, University of Helsinki, Helsinki, Finland

## Abstract

**Background:**

Longer work careers are discussed, but inequalities in health trajectories among employees facing retirement remain poorly understood. We examined social class trajectories in physical functioning among ageing female employees ten years before and after transition to old-age or disability retirement.

**Methods:**

We used Helsinki Health Study cohort data. The baseline (2000-02) included 7168 women, aged 40-60, employed by the City of Helsinki, Finland (response 67%). Follow-ups took place in 2007, 2012 and 2017 (response 78-83%). The outcome was RAND-36 Physical Functioning subscale, range 0-100, with higher scores indicating better functioning. Social classes were upper and lower class, and covariates age, work conditions and health behaviours. Mixed-effect growth curve models were used to predict functioning scores and 95% confidence intervals (CI) 10 years before and after mandatory old-age or disability retirement.

**Results:**

Old-age and disability retirees lacked class inequalities in functioning 10 years prior retirement. Towards retirement transition, functioning declined and inequalities emerged. Among old-age retirees, the predicted score was 86.1 (CI 85.2-86.9) for upper class and 82.2 (81.5-83.0) for lower class. Among disability retirees, the score was 70.3 (67.8-72.9) for upper class and 62.2 (60.4-63.9) for lower class. Among old-age retirees, functioning declined and inequalities slightly widened. Among disability retirees, the decline plateaued and inequalities narrowed. Physical work and BMI somewhat attenuated the inequalities.

**Conclusions:**

Among female employees, functioning declined and class inequalities emerged towards retirement transition. Widening inequalities were seen among old-age retirees, but not among disability retirees. Preventing the decline of functioning and related inequalities would help safeguard a healthy and successful ageing among female retirees.

**Key messages:**

• As functioning shows a constant decline before and after old-age retirement, there is a need for slowing down the pace of the decline.

• Class inequalities in functioning tend to widen among old-age retirees; egalitarian measures are needed to turn the development to narrowing inequalities.

